# How should we address the pipeline problem?

**DOI:** 10.1007/s40037-016-0266-4

**Published:** 2016-04-13

**Authors:** Hillary J. Braun

**Affiliations:** UCSF School of Medicine, University of California, San Francisco, USA

The article by Paulus et al. is the most recent in a growing area of investigation dedicated to understanding why substantial numbers of women fail to reach the highest ranks of their profession [1]. While the authors focus on gender differences in promotion in academic medicine, the medical field is one of many facing a similar problem: of 197 heads of state, only 22 are women [2]; of the top 500 companies by revenues, only 21 are headed by women [3]; in politics, women hold just 18 % of United States congressional offices [3] and 24 % of European Union membership [4]; in Hollywood, women made up only 1.9 % of the directors for the 100 top-grossing films in 2013 and 2014 [5]. There is certainly a pipeline problem, and it is not confined to medicine.

As Paulus et al. point out, traditional culprits including family responsibilities, clinical responsibilities, and teaching do not account for the gender differences in academic rank in the field of medicine. The most striking observation in their study was women’s perception of the low value of academic promotion, a finding the authors did not directly study, but postulate is the result of a combination of factors including culture, academic environment, and psychological differences such as the ‘confidence gap’.

For groups that have historically been subjected to discrimination or exclusion, a constellation of subtle factors derived from, and perpetuated by, society are thought to hinder forward progress and achievement. Examples of this include minority students who enter schools and professions in which their ethnic groups are under-represented [6], and women who experience gender discrimination in the workplace as a result of implicit biases against women [7]. I would argue that we must work towards eradicating the underlying culture that perpetuates these subtle transgressions against women in order to solve the pervasive pipeline problem, and that the ideal solution would involve an individual arm (raising awareness and empowering women to persevere through adversity) and a societal arm (critically examining and reforming society’s view of women).

An approach to individual empowerment may arise from the literature on ethnic discrimination. In 2007, Walton and Cohen described a state of ‘belonging uncertainty’, whereby minority students subconsciously monitor their learning environment for signs of lack of fit and, in turn, suffer adverse effects on their motivation and academic achievement [6]. The authors subsequently developed a ‘social belonging intervention’ where students were offered non-threatening explanations for their feelings of lack of fit and found that the academic decline nearly halved as a result of this simple intervention [8]. By reframing the way in which students perceived and internalized their environment, the authors created a measurable improvement in performance.

In the case of female physicians, who may exit the pipeline due to cultural, academic, or psychological factors, such an intervention might involve reading materials to normalize feelings of lack of fit or viewing anecdotal reports from more senior physicians with similar experiences, with accompanying writing or speaking exercises to ensure internalization of the intervention. This suggestion might raise some concern given the inevitable time constraints of an academic career, but many of these activities could be integrated in the existing infrastructure of meetings of associations of women professionals and their affiliated institutional chapters. The underlying concept of the belonging intervention is to encourage non-threatening interpretations of adversity – to say yes, you may experience this, but it has happened before and should not deter you; the reason this intervention works is because subjective interpretation of relationships or situations, as opposed to objective measures of these things, more strongly affects well-being [8].

However, priming female physicians is only one aspect of the solution; the bigger issues may be that gender-normative expectations are deeply engrained in every field from media to medicine. Today, in 2016, we continue to witness the jarring and unexpected degradation of women that ranges from an inflammatory presidential candidate in the United States, to blockbuster films that use the sexual objectification of women to generate millions in worldwide box office revenue, to rape in nearly every country and context. In addition to empowering individuals, we absolutely must reform the way society treats and portrays women.

These aforementioned explicit examples highlight a deeply rooted international issue, and while each is disturbing in its own right, I think the primary problem is that they collectively stoke the simmering flame of *implicit* gender bias – a phenomenon that arises from society’s impact on the thoughts and actions of its members. *Explicit* attitudes are conscious and largely controlled by the individual, while *implicit* attitudes are subconscious and are considered to be automatic or instinctual [9, 10]. It has been demonstrated that people can simultaneously hold opposing explicit and implicit views of the same subject [11], and that much of our behaviour is actually driven by implicit attitudes that are not under rational control [12]. The source of implicit biases is multifactorial; it is thought that biases are formed at a young age through direct and indirect exposure and are moulded throughout our lifetimes by media, news programming, and observation of who occupies valued and devalued roles in society [13]. Put concisely: if implicit bias drives behaviour and society’s visible values drive implicit bias, then bias will only be changed with a giant cultural paradigm shift – one that extends beyond an academic medical centre, a Fortune 500 company, or even a Head of State.

In a speech in 2015, United States Supreme Court Justice Ruth Bader Ginsburg said the following: “[W]hen I’m sometimes asked when will there be enough [women on the Supreme Court]? And I say ‘When there are nine.’ People are shocked. But there’d been nine men, and nobody’s ever raised a question about that.” Like many others, she too has acknowledged the pipeline problem, which cannot be distilled to a single offender and is, as the authors state, a complex problem. Perhaps, one may argue, women should see it as a duty to ascend the professional ladder regardless of their perception of the value of a promotion to normalise the position of women in power and create a legacy of mentorship and advocacy for future generations of female professionals. I agree with this belief.

However, society must change, too, so that women have the right to choose and have options about how they balance their careers and families without being penalized. Women like Facebook’s Sheryl Sandberg and Hollywood’s Patricia Arquette have used their prominent platforms to draw attention to the cultural barriers faced by women in a variety of professions, powerful media agents such as The New York Times and Harvard Business Review now publish regularly on this topic, and researchers in every field from business to medicine are pursuing a fuller understanding of where the problems lie and how we can address them. It is our duty, both as individuals and as members of a global society, to remain aware and vigilant in order to protect and expand upon the foundation that has been laid.
